# Prevalence and risk factors of gross neurologic deficits in children after severe malaria: a systematic review protocol

**DOI:** 10.21203/rs.3.rs-3374025/v1

**Published:** 2024-02-23

**Authors:** Allen Eva Okullo, Chandy C. John, Richard Idro, Andrea L. Conroy, Alison Annet Kinengyere, Kevin Ouma Ojiambo, Caroline Otike, Simple Ouma, Moses Ocan, Ekwaro A. Obuku, Michaël Boele van Hensbroek

**Affiliations:** University of Amsterdam Faculty of Medicine: Amsterdam UMC Locatie AMC; Indiana University School of Medicine; Makerere University Faculty of Medicine: Makerere University College of Health Sciences; Indiana University School of Medicine; Sir Albert Cook Memorial Library: Makerere University College of Health Sciences; Makerere University Faculty of Medicine: Makerere University College of Health Sciences; JCRC HQ - Lubowa: Joint Clinical Research Center; Makerere University Faculty of Medicine: Makerere University College of Health Sciences; Makerere University Faculty of Medicine: Makerere University College of Health Sciences; London School of Hygiene & Tropical Medicine; University of Amsterdam Faculty of Medicine: Amsterdam UMC Locatie AMC

**Keywords:** Gross neurologic deficit, cerebral malaria, severe malaria, prevalence, risk factors, children

## Abstract

**Background:**

Children exposed to severe malaria may recover with gross neurologic deficits (GND). Several risk factors for GND after cerebral malaria (CM), the deadliest form of severe malaria, have been identified in children. However, there is inconsistency between previously reported and more recent findings. Although CM patients are the most likely group to develop GND, it is not clear if other forms of severe malaria (non-CM) may also contribute to the malaria related GND. The aim of this systematic review is to synthesize evidence on the prevalence and risk factors for GND in children following CM and map the changes in patterns over time. In addition, this review will synthesize evidence on the reported prevalence and risk factors of gross neurologic deficits following other forms of severe malaria.

**Methods:**

The systematic review will be conducted according to recommendations of the Preferred Reporting Items for Systematic Reviews and Meta-Analyses for Protocols (PRISMA-P). Relevant research articles will be identified using relevant search terms from the following databases: MEDLINE, Embase, Web of Science and Global Index Medicus (GIM). The articles will be screened at title and abstract, then at full text for inclusion using *a priori* eligibility criteria. Data extraction will be done using a tool developed and optimized in Excel spreadsheet. Risk of bias assessment will be done using appropriate tools including ROBINS-E (‘Risk Of Bias In Non-randomized Studies of Exposure’) tool, while publication bias will be assessed using funnel plot. A random-effects meta-analysis and structured narrative synthesis of the outcomes will be performed and results presented.

**Discussion:**

Findings from this systematic review will inform policy makers on planning, design and implementation of interventions targeting the treatment and rehabilitation of GND following severe malaria in children.

**Systematic review registration:**

The protocol is registered in the International Prospective Register of Systematic Reviews (PROSPERO), registration number CRD42022297109.

## BACKGROUND

In 2021 an estimated 247 million cases of malaria were reported from 84 malaria endemic countries globally, with the WHO African region accounting for 95% of this burden [1]. The severe malaria burden is estimated at less than 1% of the total malaria cases diagnosed [2] with under-five-year-old children in sub-Saharan Africa being most at risk of both severe malaria and death from severe malaria [3].

Severe malaria is a multi-syndromic illness complication of plasmodium falciparum infection that manifests in form of cerebral malaria, severe malaria anemia, respiratory distress, malaria with seizures and malaria with prostration among under five-year-old children [3]. Survivors of cerebral malaria (CM), may develop neurological sequelae, some of which may cause life-long disabilities [3–7]. These sequelae may include gross neurologic deficits (GND) such as cortical blindness, ataxia, hemiparesis, deafness and abnormal movement disorders [3–5] and are often more common among children, with the prevalence ranging from 6 to 29% at hospital discharge [3–5, 8, 9]. Survivors with GND may die within a few months after discharge [4].

Several studies have reported significant declines in the proportion of children with persistent neurological sequelae after CM in the first months’ post-discharge [8, 10–12]. In a study conducted in The Gambia [8], the proportion of children with neurological sequelae after CM had reduced from 23.3% at discharge to 8.6% one month after discharge, with a further decline to 4.4% at six months. Similarly, in Uganda, the proportion of children with neurological sequelae after CM declined from 28.2% at discharge to 9.5% at three months after discharge, with all children recovering by six months post-discharge [11].

Some neurologic sequelae, like cortical blindness, may be transient [13–16], while others may persist, including hemiplegia, paresis, extrapyramidal features, and epilepsia [4, 16]. Consequences of these sequelae include a reduced quality of life and an increase in disability-adjusted life years [16].

Risk factors for neurological sequelae following severe malaria among children include deep and prolonged coma, multiple seizures [3–5, 8, 9], acute seizures [7], intracranial hypertension [8, 12, 17–19], male gender, a higher admission temperature [20]; age younger than 3 years [17], acute kidney injury (AKI) [21–23] and a biphasic clinical course marked by recovery of consciousness followed by recurrent convulsions and coma [12]. Also, the antimalarial artesunate has previously been associated with ataxia and slurring of speech [24], however new evidence, on the contrary, suggests fewer neurologic deficits in children receiving these artemisinin-based treatments [25].

### Why this review?

Recent studies have not been able to confirm the importance of previously associated factors such as hypoglycemia, anemia, age, sex, multiplicity of convulsions, and artesunate treatment with persistence of GND after CM [5, 8, 9, 25]. There is need for evidence on whether this is a true change over time or if it is due to a difference in reporting patterns or analysis. In addition, no systematic review has synthesized evidence on the prevalence or risk factors of GND among children after CM or non-CM severe malaria which is far more common than CM. It is crucial that the prevalence of GND among different forms of severe malaria and the most important risk factors are identified in this synthesis to inform the targeting and development of effective treatment protocols to prevent GND among children.

This systematic review will synthesize the prevalence and risk factors for GND in children with CM; the prevalence and risk factors for GND in children with non-CM; and map changes in prevalence of GND during follow-up after the episode of CM or of non-CM. This review will also look at the age-related risk factors in addition to comprehensive evidence on all risk factors for GND after CM and non-CM, which has not been done before.

## METHODS

This systematic review protocol has been written following the Preferred Reporting Items for Systematic review and Meta-Analysis Protocols 2015 guidelines (PRISMA-P) [26]. The protocol is registered in the International Prospective Register of Systematic Reviews (PROSPERO), registration number CRD42022297109.

### Review question

#### Primary review question

What is the prevalence of gross neurologic deficits among children after CM and non-CM in malaria affected regions globally?

#### Secondary review question

What are the risk factors for gross neurologic deficits among children after CM and non-CM in malaria affected regions globally?

### Eligibility criteria

Peer-reviewed articles will be selected according to the following criteria.

### Inclusion criteria:

- Peer-reviewed articles reporting on GND after WHO-defined severe malaria. GND refers to an abnormal neurologic function of a specific body part arising from injury to the brain, spinal cord, muscles or nerves that feed the affected area.
- Peer-reviewed articles reporting on GND after severe malaria from the earliest published study in 1946 to date.
- Peer-reviewed articles reporting on risk factors for GND after severe malaria. Risk factors will include multiple seizures, deep/prolonged coma, hypoglycemia, clinical features of intracranial/hypertension, anemia, age, sex, malnutrition (macronutrient and micronutrient deficiency), hyperpyrexia, acute kidney injury, chronic kidney disease, hemolysis, endothelial activation, central nervous system inflammation, parasitic biomass, endothelial dysregulation, antimalarials like artesunate.
- Peer-reviewed articles reporting on children (persons younger than 18 years).
- Peer-reviewed articles with cross-sectional, case-control, cohort, randomized controlled trials, quasi-experimental study, and case series designs.
- Peer-reviewed articles in all languages. Google translator will be used for articles not in English. These will qualify at title and abstract screening.

### Exclusion criteria:

- Peer-reviewed articles which are: editorials, opinions, consensus papers, conference abstracts, guidelines, recommendations, qualitative research, and literature reviews.
- Peer-reviewed articles whose full text cannot be retrieved. The librarian and information specialist AAK will search for full text articles of all included peer reviewed studies from external sources such as Web of Science, EMBASE and Lib-Hub and in consultation with other librarians. Articles whose full texts are not accessed despite these efforts, will not be included.

The review will be guided by the following elements of population, intervention/exposure, comparator, study design, setting and timing (PICOST) as shown in Table 1.

### Patient and public involvement

There will be no patient involvement.

### Population

The target population for this review are children (persons below 18years) who had any form of severe malaria. The forms of severe malaria will include: cerebral malaria, severe malaria anemia, respiratory distress, malaria with seizures and malaria with prostration.

### Exposures

Exposures will include different forms of severe malaria such as cerebral malaria, severe malaria anemia, respiratory distress, malaria with seizures and malaria with prostration.

### Comparator

This review will not have a comparator given that we are focusing on prevalence and risk factors for GND among children who had different forms of severe malaria.

### Outcomes

The primary outcome of this review will be prevalence of GND after severe malaria among children. Gross neurologic deficits will include motor impairments and movement disorders (ataxia, tremor, dystonia, cranial nerve palsies, monoparesis, hemiparesis or quadriparesis and monoplegia, hemiplegia and quadriplegia), speech and language impairments, hearing and visual impairment. The secondary outcome of this review will be risk factors for GND among children who had different forms of severe malaria. Risk factors may include: multiple seizures, deep/prolonged coma, hypoglycemia, clinical features of intracranial hypertension, anemia, age, sex, malnutrition, hyperpyrexia, acute kidney injury, chronic kidney disease, hemolysis, endothelial activation, central nervous system inflammation, parasitic biomass, micronutrient deficiency, endothelial dysregulation, antimalarials such as artesunate.

### Setting

This review will include studies from all malaria affected regions globally.

### Timing

This review will include studies from the earliest date of publication on severe malaria (1946) to date.

### Language

There will be no language restriction to this review.

### Information sources

The following electronic databases will be searched: MEDLINE, Embase, Web of Science and Global Index Medicus (GIM). In addition to this, we will search bibliographies of all included studies. We will search for grey literature from the WHO website, institutional repositories and from leading researchers on neurologic deficits after severe malaria.

### Search strategy

This review will use a search strategy developed in collaboration with an experienced librarian and information specialist (AAK) skilled in systematic reviews. AAK will perform the article search using search terms together with their synonyms and MeSH (Medical subject Headings) developed from our review of articles published in peer-reviewed journals on neurological sequelae after severe malaria from the earliest published study to date. The following search terms will be used to identify eligible studies: ‘child’, ‘children’, ‘pre-school’, ‘infant’, ‘infants’, ‘malaria’, ‘cerebral’, ‘severe malaria’, ‘impaired consciousness’, ‘acidosis’, ‘respiratory distress’, ‘multiple convulsions’, ‘prostration’, ‘repeated seizures’, ‘multiple seizures’, ‘hypoglycaemia’, ‘severe anaemia’, ‘severe anemia’, ‘renal impairment’, ‘acute kidney injury’, ‘jaundice’, ‘pulmonary oedema’, ‘significant bleeding’, ‘abnormal bleeding’, ‘shock’, ‘hyperparasitaemia’, ‘hyperlactataemia’, ‘nervous system disease’, ‘brain disease’, ‘neurologic’, ‘sequelae’, ‘complication’, ‘impairment’, ‘deficit’, ‘prognosis’, ‘risk factors’, ‘prevalence’, ‘frequency’, ‘risk’, ‘prediction’, ‘epidemiological’, ‘association’. The terms will be combined using Boolean operators “OR”, or “AND” and truncation where applicable.

A pilot search conducted in MEDLINE will be tested for precision to ensure a high proportion of appropriate articles are retrieved. Our initial screening of an electronic search of 755 titles and abstracts performed on 6 September 2023, in MEDLINE yielded 37 (4.9%) articles as potentially eligible (Table 2). The search string from MEDLINE has been included as an additional file. The final validated search string from MEDLINE will be adapted to the syntax of other databases, that is, Embase, Web of Science, and Global Index Medicus, and these will also be searched as part of the review. The identified articles will be transferred to EPP-reviewer software.

### Selection process and data management

All identified references will be imported into EPPI-reviewer (eppi.ioe.ac.uk/EPPIReviewer-Web) with full texts and bibliography details. We shall then run a duplicate search and remove all duplicates. A screening tool will be developed based on the study inclusion and exclusion criteria. This will be tested for its robustness on 5% of the articles, among four reviewers. Titles and abstracts will be screened for potential relevance by four independent reviewers (AEO, KOO, CO, and SO) in duplicate, that is, AEO and KOO, CO and SO, AEO and CO, KOO and SO, AEO and SO, KOO and CO, using *a priori* inclusion and exclusion criteria. Any disagreements between the reviewers will be resolved through consensus after discussion. The selected articles will then be screened at full text independently by the four reviewers working in duplicate. Any disagreements will be resolved by discussion. Screening and coding will be done using EPPI-reviewer software. The total number of studies included and excluded at each stage of the selection process will be presented in a PRISMA flow chart, while the reasons for exclusion or inclusion will be presented in the main text.

### Data abstraction

A data abstraction tool will be developed in EPPI-Reviewer and pre-tested using 5% of the selected articles. The data abstraction tool will include key variables for abstraction from each article, namely: study characteristics (author, year of publication, year of data collection, title, citation, institution, country, language, source of funding), study design, population characteristics (sample size, age at diagnosis of severe malaria), type of gross neurologic deficit (including author specific definitions), duration of gross neurologic deficit, type of assessment for gross neurologic deficit, risk factors, measures of association (such as risk ratios, odds ratios), prevalence of gross neurologic deficit. The reviewers (AEO, KOO, CO, SO) will independently abstract the data, and any disagreements will be resolved through discussion.

### Preliminary findings

In order to determine feasibility of this review, we have listed five studies from the MEDLINE search that meet the eligibility criteria after initial screening of titles and abstracts (Table 3).

These studies were published between 1990 and 2013 and were conducted among children who had previously been admitted in hospitals in Kenya [5], The Gambia [8, 12], and Nigeria [9, 14]. These studies reported prevalence and risk factors for neurologic sequelae after childhood cerebral malaria. GND within these studies ranged from 12–23.8% which is the primary outcome of the study. Risk factors for GND from these studies included prolonged coma, depth of coma, multiple convulsions, hypoglycemia, previous seizures, multiple seizures, and focal neurological signs observed during admission. Risk factors are the secondary outcome of the study.

### Risk of bias assessment

Four members of the team (AEO, KOO, CO and SO) shall independently assess the methodological quality of included studies. Risk of bias assessment shall be done using ROBINS-E (‘Risk Of Bias In Non-randomized Studies- of Exposures’) tool for non-randomized controlled trials (https://www.riskofbias.info/welcome/robins-e-tool). Using the ROBINS-E, bias will be assessed as a judgement (low risk of bias, some concerns, high risk of bias, very high risk of bias) using seven domains. These domains are: risk of bias due to confounding; risk of bias arising from measurement of the exposure; risk of bias in selection of participants into the study (or into the analysis); risk of bias due to post-exposure interventions; risk of bias due to missing data; risk of bias arising from measurement of the outcome; risk of bias in selection of the reported result.

The Cochrane Risk of Bias Tool will be used for randomized control trials (RCTs) (https://methods.cochrane.org/bias/resources/cochrane-risk-bias-tool). Using this tool, bias will be assessed as a judgement (high, low or unclear) for individual elements using five domains namely: selection bias; reporting bias; performance bias; attrition bias; detection and other biases [27]. Other study designs will be assessed using the Qualitative Assessment Tool for Quantitative Studies (Effective Public Health Practice Project, https://merst.ca/ephpp/). This tool provides an overall rating (weak, moderate or strong) based on an appraisal of eight domains: selection bias, study design, confounders, blinding, data collection methods, withdrawals and dropouts, intervention integrity and analysis.

### Publication bias

We will assess the risk of publication bias in the included articles by using the asymmetry of funnel plots. This is a rank-based data augmentation technique which has proved to be accurate for assessing publication bias due to missing data or studies [28]. In the absence of missing studies, the shape of the scatter plot should resemble a symmetrical inverted funnel with a wide base (consisting of small studies with large effect estimate variability) and a narrow top (consisting of large studies with small effect estimate variability) [29]. The presence of large ‘holes’- often seen close to the bottom- or asymmetry in the plot indicates publication bias, though these holes may have other causes, such as study heterogeneity [30].

### Assessment of strength and confidence of cumulative evidence

Strength of evidence will be graded based on the assessment of study limitations, directness, consistency, precision, and reporting bias. The overall quality of evidence will be assessed using a modified Grading, Recommendation, Assessment Development and Evaluation (GRADE) framework. In the GRADE method, the quality of evidence is rated for each key outcome as ‘High’, ‘Moderate’, ‘Low’ or ‘Very Low’. Observational studies start at low quality and may be upgraded, whilst randomized trials are set at high quality and may be downgraded. We will develop a summary of findings tables and assess the confidence in the effect estimates and strength of associations based on the determined quality of evidence.

### Heterogeneity

The I^2^ statistic will be used to assess the level of statistical heterogeneity in the articles. The I^2^ statistic will show the percentage (%) of heterogeneity attributable to between-study variation [31]. Heterogeneity will be categorized as, low (I^2^ = 25%) (low), moderate (I^2^ = 50) and high (I^2^ > 75%) [27]. Sub-group analysis will only be done among articles categorized as low and moderate heterogeneity.

### Missing data

We will denote variables which are desired but missing or not reported as “NR” and seek clarification by contacting the corresponding authors. In the event that a corresponding author cannot be assessed, we will only report the characteristics of the study and not proceed to do a meta-analysis. We will not employ any statistical methods for handling missing data.

### Data synthesis

Prevalence values as well as risk ratios and odds ratios for binary outcome variables will be synthesized separately. Effect sizes will be pooled statistically using inverse variance weighted random effects meta-analysis, using the metan command in Stata v16. Pooled effects will be expressed in metric that is relevant, for example, a percentage change in risk measured in standard units of outcome.

Synthesis will further be in form of summary of findings tables and forest plots where applicable using STATA version 16. This will follow the format of the Cochrane consumers and communication review group [32]. Outcome data will be presented as frequencies and percentages. Prevalence values and measures of association such as risk ratios and odds ratios, will be presented as appropriate. We will organize and tabulate results to identify associations which will be described narratively. We will describe the included articles, group articles according to study design, exposure and outcome. We shall use both narrative and quantitative synthesis.

### Sensitivity analysis

Sensitivity analysis will be done by removing studies from the meta- analysis one-by-one to see if the results of the meta-analysis are sensitive to any single study. We will also examine sensitivity of findings to risk of bias status (low risk, some concerns and high risk).

## DISCUSSION

### Proposed use of results

This systematic review will synthesize evidence on prevalence and risk factors for GND after all forms of severe malaria among children. This will provide information on the burden of GND among children after different forms of severe malaria which will guide policy makers on targeting interventions for children at the highest risk of GND after severe malaria. This systematic review will also provide clarity on the most important risk factors for GND after severe malaria in children which will inform the selection, design, deployment and establishment of interventions targeting early detection, prevention and rehabilitation of GND following severe malaria among children. This systematic review will thus provide comprehensive evidence to inform policymakers and clinicians in the control and rehabilitation efforts for GND associated with severe malaria among children.

### Anticipated methodological limitations

There may be a limitation in identifying all relevant studies on prevalence and risk factors for GND among children after severe malaria due to grey literature beyond the reach of the review team. This will be addressed by use of publication bias which will aid interpretation of the study findings.

## Figures and Tables

**Table 1:** Review question using the PICOST framework

Population	Children (persons younger than 18years) who had severe malaria
Exposure	Different forms of severe malaria such as cerebral malaria, severe malaria anemia, respiratory distress, malaria with seizures and malaria with prostration
Comparator	None
Outcome	Primary outcome: prevalence of GND such as motor impairments and movement disorders (ataxia, tremor, dystonia, cranial nerve palsies, monoparesis, hemiparesis or quadriparesis and monoplegia, hemiplegia and quadriplegia), speech or language impairments, hearing or visual impairment
	Secondary outcome: risk factors such as multiple seizures, deep/prolonged coma, hypoglycemia, clinical features of intracranial hypertension, anemia, age, sex, malnutrition (macro and micronutrient deficiency), hyperpyrexia, acute kidney injury, chronic kidney disease, hemolysis, endothelial activation, central nervous system inflammation, parasitic biomass, endothelial dysregulation, antimalarials like artesunate
Study design	The review will include cross-sectional studies, cohort studies, case control studies, randomized controlled trials, diagnostic studies, quasi experimental study designs, and case series
Setting	Malaria affected regions globally
Timing/Date of Publication	1946 (earliest date for the first journal publication on severe malaria) to date

**Table 2:** Feasibility of yield of literature of pilot electronic search for prevalence and risk factors of gross neurologic deficits among children after severe malaria in MEDLINE

Search number (Database)	Search terms (and date)	Number of hits^a^ (Relevant)
#1 MEDLINE	exp child/ or (children or pre-school or infant or infants).ti,ab,kf. AND exp Malaria, Cerebral/ or ((cerebral adj3 malaria) or severe malaria or impaired consciousness or acidosis or respiratory distress or multiple convulsions or prostration or repeated seizures or multiple seizures or hypoglycaemia or severe anaemia or severe anemia or renal impairment or acute kidney injury or jaundice or pulmonary oedema or significant bleeding or abnormal bleeding or shock or hyperparasitaemia or hyperlactataemia).ti,ab,kf. AND (“nervous system disease*” or “Brain Disease*” or (“neuro* complicat*” or “neuro* impair*” or “neuro* deficit*” or “neuro* sequelae”)).ti,ab,kf. AND exp Prognosis/ or exp Risk Factors/ or prevalence/ or (prevalence or frequen* or risk or prognos* or predict* or epidemiological or associat*).ti,ab,kf.	^a^755 (^b^37, 4.9%)

a Number of article titles and abstracts as at 6 September 2023

b Sorted by relevance and initial screening of titles and abstracts

**Table 3:** Preliminary findings of potentially eligible studies

Author	Year	Country	Design	Population
Idro et al [5]	2006	Kenya	Cross sectional	Children
Van Hensbroek et al [8]	1997	The Gambia	Prospective cohort	Children
Oluwayemi et al [9]	2013	Nigeria	Cross sectional	Children
Brewster et al [12]	1990	The Gambia	Prospective cohort	Children
Bondi [14]	1992	Nigeria	Prospective cohort	Children

## Data Availability

This section is not applicable since this is a systematic review protocol with no results yet.

## Abbreviations

GND: Gross Neurologic Deficit
GRADE: Grading, Recommendation, Assessment, Development and Evaluation
PICOST: Population, Intervention/Exposure, Comparator, Outcome, Study design, Timing
PRISMA-P: Preferred Reporting Items for Systematic reviews and Meta-Analysis Protocols
PROSPERO: International Prospective Register of Systematic Reviews
RCT: Randomized Controlled Trials
ROBINS-E: Risk of Bias in Non-randomized Studies of Exposures
